# The functional microbiota of on- and off-year moso bamboo (*Phyllostachys edulis*) influences the development of the bamboo pest *Pantana phyllostachysae*

**DOI:** 10.1186/s12870-022-03680-z

**Published:** 2022-06-24

**Authors:** Jian Li, Qing Zhao, Jin-Peng Huang, Jia-Yu Jia, Teng-Fei Zhu, Tao Hong, Jun Su

**Affiliations:** 1 College of Forestry, Fujian Agriculture and Forestry University, Fuzhou, 350002 China; 2 Key Laboratory of Forest Ecosystem Process and Management of Fujian Province, Fuzhou, 350002 China; 3 Basic Forestry and Proteomics Research Center, Fujian Provincial Key Laboratory of Haixia Applied Plant Systems Biology, Fujian Agriculture and Forestry University, Fuzhou, 350002 China; 4 Department of Molecular, Cell & Developmental Biology, University of California, Los Angeles, CA 90095 USA

**Keywords:** Moso bamboo (*Phyllostachys edulis*), Biennial cycle, *Pantana phyllostachysae*, Metagenomics, Gut microbiota

## Abstract

**Background:**

Development of *Pantana phyllostachysae*, a moso bamboo pest, is affected by its diet. Understanding the mechanism underlying the different insect-resistant capacities of on- and off-year moso bamboo fed by *P. phyllostachysae* is crucial for managing pest outbreaks. As microbes were proven to influence plant immunity, we compared gut microbial communities of *P. phyllostachysae* with different diets by metabarcoding sequencing. By using sterilization assay, microbes were removed from leaf surfaces, and thus we confirmed that microbes inhabiting moso bamboo leaves impact the weight of *P. phyllostachysae* larva. Furthermore, the gut microbial communities of *P. phyllostachysae* fed on on- and off-year bamboo leaves were compared, to identify the functional microbial communities that impact the interaction between bamboo leaves and *P. phyllostachysae*.

**Results:**

We found that species from orders Lactobacillales and Rickettsiales are most effective within functional microbiota. Functional prediction revealed that gut microbes of larva fed on on-year leaves were related to naphthalene degradation, while those fed on off-year leaves were related to biosynthesis of ansamycins, polyketide sugar unit biosynthesis, metabolism of xenobiotics, and tetracycline biosynthesis. Most functional microbes are beneficial to the development of larva that feed on on-year bamboo leaves, but damage the balance of intestinal microenvironment and immune systems of those larva that feed on off-year leaves.

**Conclusions:**

This work developed an efficient strategy for microbiome research of Lepidopteran insects and provided insights into microbiota related to the interaction between host plants and *P. phyllostachysae*. We provided microbial candidates for the ecological control of *P. phyllostachysae* according to the function of effective microbiota.

**Supplementary Information:**

The online version contains supplementary material available at 10.1186/s12870-022-03680-z.

## Background

Moso bamboo (*Phyllostachys edulis*) is well known for its rapid growth rate and high carbon sequestration value, as well as its fast-increasing planting area (approximately 3% annually) due to its high economic value [1]. Development of moso bamboo strict to the on- and off-year biennial cycle [2]. The plants produce more shoots in ‘on-year’ but gain more timber volume (potential yield) in ‘off-year’ [3]. Intensive forest management (IFM) is required in commercial moso bamboo forests to maximum economic benefits, which destroyed the on- and off-year bamboo distribution and caused on-year bamboo to dominate the field [4, 5]. The diversity of arthropod communities differs between on- and off-year bamboo leaves, with the population of phytophagous insects (typically *Pantana phyllostachysae*, the most destructive moso bamboo lepidopteran defoliators) being significantly higher on on-year bamboo leaves, which weakens the insect-resistant capacity of commercial moso bamboo forests (more severe and frequent defoliator damages) compared to ecological forest [6, 7]. Significant biochemical and molecular ecological research on the connections between moso bamboo and *P. phyllostachysae* have been conducted to understand how IFM weakens the insect-resistant capacity of moso bamboo forests. These studies found that differences in morphology, secondary metabolism, and nutritive element content created the differences in the capacity for insect resistance of on- and off-year moso bamboo [6, 8]. Also, the internal aging signal regulated the amount of *PhSPL17* (*Phyllostachys heterocycla* SQUAMOSA promoter binding protein-like 17), which negatively impacted the jasmonate response of on-year bamboo leaves, leading to the tolerance of *P. phyllostachysae* that feed on on-year leaves. It thus reshaped the diversity of arthropod communities via bottom-up effects [9–12]. However, other than biochemical and genetic changes, the differences of the insect-resistant systems of on- and off- year moso bamboo remain unknown. Understanding these differences is crucial to explore the mechanism of pest outbreaks in commercial moso bamboo forests [9].

Plants form their own defense systems by co-evolving with insects; the morphological structure of leaves, such as thorns, fur, and cuticle, are fundamental in this co-evolution process [13]. In addition, plants can influence the tropism and feeding activities of phytophagous insects through their secondary metabolites, which in turn affects the growth, development, and reproduction of insects and causes their populations to fluctuate [14, 15]. Increasing amounts of evidence have shown that microbes are crucial components of plant defense systems against insects, and they have a significant impact on the interaction between plants and insects. Microbes can regulate plant secondary metabolites and indirectly induce the production of volatile organic compounds (VOCs), which are closely related to insect feeding behavior and oviposition stimulation. In this way, they can change insect biological functions, including metabolism and behavior. In addition, both VOCs and plant-hosted microorganisms can drive the structural changes of insect symbiotic microbes (especially gut microbiome) by feeding behavior. In this way, it affects the development of herbivorous insects [16]. The interaction between the symbiotic microorganisms of plants and herbivorous insects can reveal the plant defense mechanisms and their internal mechanisms of action. However, it also can be seen that the influence of microorganisms on the relationship between plants and insects is extremely complicated, which is a great challenge for research on forest microorganisms [17]. Hence, most of the practical significance for forestry research is identification of those functional microbes, which may be developed into pest control agents suitable for application in the field [18]. Our previous studies on *Beauveria bassiana*, a fungus that is a natural enemy of *P. phyllostachysae,* found that continuous feeding on on-year bamboo leaves is conducive to the survival and development of *P. phyllostachysae*, but limits the survival of *B. bassiana* [10, 11]. By profiling the gut microbiomes of nine bamboo-feeding insects (including Lepidoptera species), we found that microbial communities may affect the development of insects to adapt to their feeding behaviors [19]. These findings suggest that feeding behavior can affect the development of *P. phyllostachysae* though gut microbes.

Although the use of metagenomic sequencing has promoted high-throughput profiling of the microbial community, the identification of functional microbes from microbiota is still difficult due to the lack of an effective large-scale screening strategy [20–22]. Previous works have shown that manipulating artificial assemble microbial community is an effective strategy to identify functional microbes [23]. However, most artificial assembly approaches require a clear understanding of potential targets and are not suitable for high-throughput screening [23, 24]. In this study, we hijacked antibiotics capable of eliminating both bacteria and fungi on the bamboo leaves to randomly assemble an artificial microbial community [25]. We aim to develop an effective way to perform large-scale screening of functional microbes in insects.

In conclusion, further exploration of the whole picture of microbial features formed by bamboo-insect interaction is important to uncovering the mechanism underlying pest outbreaks, and it may provide new ideas for the ecological control of pests in bamboo forests. To understand the microbiota of on- and off-year moso bamboo leaves, the gut microbial constructs of *P. phyllostachysae* that feed on different types of bamboo leaves were compared via metagenomic sequencing. This study provides new insight on how different types of bamboo leaves affect the development of phytophagous insects though the microbial community. Moreover, a novel leaf sterilization assay was conducted to narrow down the functional microbiota which promote resistance to *P. phyllostachysae.* The results of this study will be helpful to further understand the mechanism of *P. phyllostachysae* outbreaks in moso bamboo forests, as well as for providing new microbial pesticide candidates to effectively control *P. phyllostachysae* [26].

## Methods

### Plant materials and treatment

The morphological traits of on- and off-year moso bamboo leaves were described in our previous work [10]. Different types of moso bamboo leaves were havested by Jian Li in Fuzhou, Fujian province, China (26° 05′ 20″ N, 119° 13′ 45″ E), once a day for further insect feeding and sterilization assay. The necessary field work permits were obtained from Fujian Agriculture and Forestry University.

### Insect feeding assay

*Pantana phyllostachysae* were collected by Jun Su in same location where plant materials were harvested, and insects were cultured as described previously [9, 10]. Permission to collect insect samples was obtained from Fujian Agriculture and Forestry University. On- and off-year moso bamboo leaves that directly collected from field were used as the diet of the larvae, with *n* = 15 individuals in each replicate and three replicates per treatment [9, 10]. The weights were recorded for the 3rd instar larvae. The insects were reared in the laboratory at 25 °C and 70% relative humidity under a 14 h-light/10 h-dark cycle. All of the leaves were fed to the insects on the second hour of the light stage of the cycle.

### Leaf sterilization assay

The microbes on the bamboo leaves were removed by incubation with 1/1000 antibiotics-antimycotic (A6533, Macklin Inc., Shanghai, China) for 4 h. The leaves were then washed three times with sterilized ddH_2_O and dried on a clean bench for 2 min. These served as post-sterilization moso bamboo leaves. Moso bamboo leaves harvested directly from fields were incubated with sterilized ddH_2_O for 4 h and dried on a clean bench for 2 min. These served as pre-sterilization moso bamboo leaves (control). The pre- and post-sterilization leaves with a similar leaf area (10.2 ± 1.1 cm^2^) were fed to 3rd instar *P. phyllostachysae* larvae for further screening of functional microbiota.

### Metagenomic sequencing

#### Sample collecting

On- and off-year bamboo leaves were collected direct from the field and kept in liquid nitrogen, as well as the combinate sterilized bamboo leaves, with *n* = 5 individuals for each type. The guts of 3rd instar larvae of *P. phyllostachysae* that fed on on- and off-year bamboo leaves were harvested and crashed in liquid nitrogen, with *n* = 15 individuals for each treatment [9].

#### DNA extraction

Total genomic DNA from samples of leaves and guts were extracted using MoBio PowerSoil DNA Isolation Kit (12855-50, MoBio, USA) according to the manufacturer’s protocol. DNA quantity and quality were measured on a NanoDrop 2000 spectrophotometer (Thermo Fisher Scientific, USA). DNA integrity after extraction was determined using 1% agarose gel. The extracted DNA was stored at − 80 °C.

#### PCR amplification and high-throughput sequencing

The V3-V4 variable region of the bacterial 16S rRNA was amplified with primers 338F (5′-ACTCCTACGGGAGGCAGCAG-3′) and 806R (5′-GTGGACTACHVGGGTWTCTAAT-3′), as well as the ITS1 rRNA gene with primers ITS1F (5′-CTTGGTCATTTAGAGGAAGTAA-3′) and ITS2R (5′-GACTACHVGGGTATCTAATCC-3′). The amplify process was described previously, via the Illumina MiSeq platform for sequencing with PE300 model at Allwegene Company (Beijing, China) [9].

#### Statistical and bioinformatics analysis

The 16S and 18S rRNA gene sequences were processed and demultiplexed through the open-source software pipeline QIIME2 (Version 1.8.0). Quality filter and trim, denoise (error-correction), pair-end read merge, chimeric remove, and taxonomy assignment were processed by Vsearch (Version 2.7.1). The subsequent data processing and analysis was described previously [27]. Differences were considered significant when *P < 0.05 and extremely significant when **P < 0.01. IBM SPSS, version 22.0 (IBM, Chicago, United States) software was applied for statistical analysis.

We used clean tags to cluster (or reduce noise) to generate operational taxonomic units (OTUs). The clustering method can be selected as uparse, uclust, or ref. reference database with the default being uparse; the noise reduction method was unoise3method [28–30]. Statistical analyses were performed at the amplicon sequence variant (ASV) level, a higher-resolution equivalent of OTU. The Shannon rarefaction curves and other richness and diversity indices of bacterial community (i.e., chao1, observed_species, and PD_whole_tree) were estimated using the QIIME2 (Version 1.8.0) platform. One-way Anova (Tukey’s test) was performed to determine the differences among groups. Rarefaction curve figure was performed using R software (Version 4.1.1). The Bray–Curtis similarity index was used as a metric of similarity between the microbial communities based on the abundance of ASVs between samples [31]. Putative microbiota functions were predicted by annotating pathways of ASVs against the Cluster of Orthologous Groups (COG) database using PICRUST2 (Version 2.0.0) [32]. A heat map and accumulation histogram were generated with R software (Version 4.1.1).

## Results

### Microbes led to differences of *Pantana phyllostachysae* via feeding on on- and off-year moso bamboo leaves

**Fig. 1:**
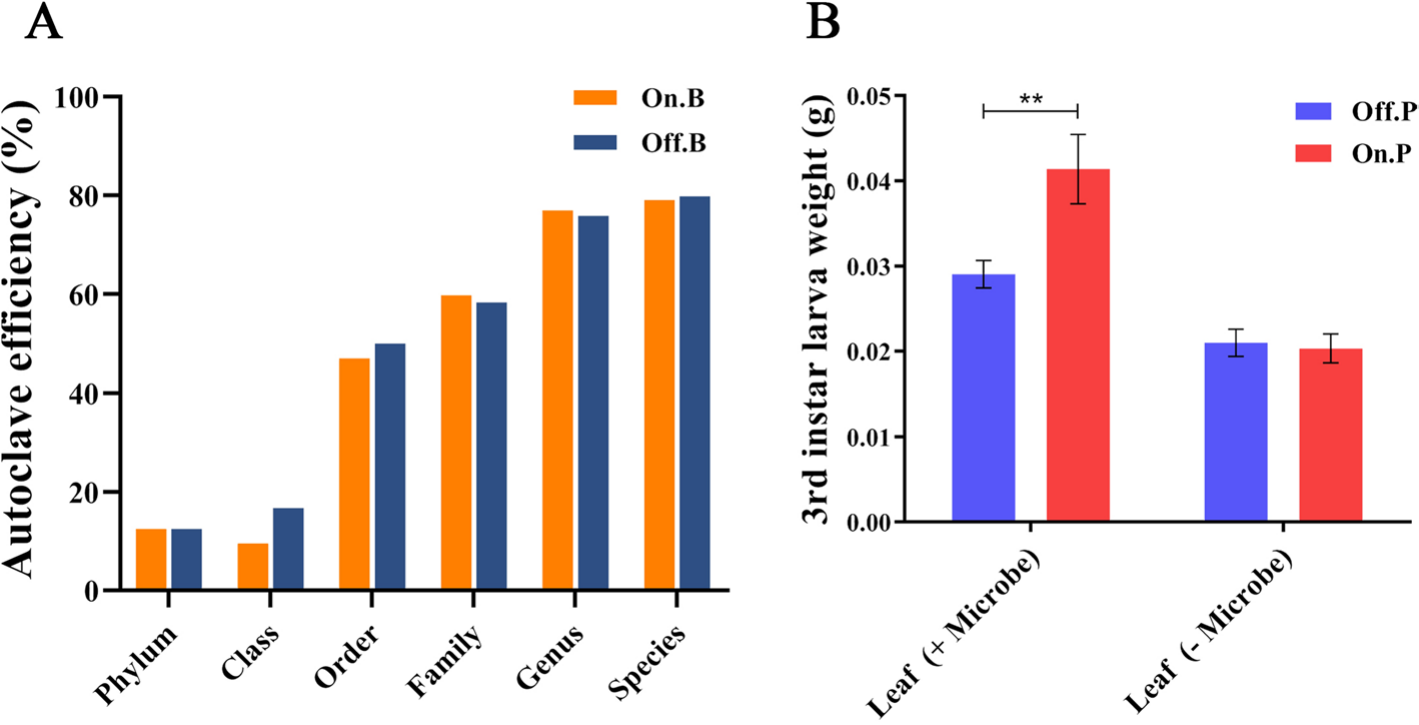
Microbes responsible for differences of *Pantana phyllostachysae* fed on on- or off-year moso bamboo leaves. **A** The sterilization efficiency of On.B (on-year bamboo leaves) and Off.B (off-year bamboo leaves) on different taxonomical level were plotted by the ratio of (number of OTUs of survived microbes) / (number of OTUs of microbes before autoclaving). **B** 3rd instar larva of different *P. phyllostachysae* populations, On.P (*P. phyllostachysae* feeding on on-year bamboo leaves) and Off.P (*P. phyllostachysae* feeding on off-year bamboo leaves), post- (− Microbe) and pre-sterilization (+Microbe), bamboo leaves, were weighed. Each box represents a mean value, with bars representing one standard deviation (SD). ** indicate statistical differences (*P* < 0.01) between samples using one-way ANOVAs (Tukey’s test)

The microbiota of moso bamboo leaves were sequenced, including 529 OTUs, belonging to 17 phyla, 50 classes, 83 orders, 134 families, and 188 genera. The Shannon index revealed that the microbial community of on-year bamboo leaves (mean = 1.43) was more diverse than that of off-year (mean = 1.14) (Fig. S1 A). Also, obvious differences of microbial community structure were found between different types of bamboo leaves (Fig. S1 B, C). To further explore the role of microbes in the pest resistance of moso bamboo, we developed a leaf sterilization assay to randomly eliminate the microbes inhabiting bamboo leaves. The efficiency of the sterilization assay was measured by comparing the microbial community structure of bamboo leaves with or without treatment (Fig. S2). After sterilization, 24% of total genera, 41% of total families, 50% of total orders, and 88% of total phyla were eliminated from the microbial community of different types of bamboo leaves (Fig. 1A). By controlling the diet of *P. phyllostachysae*, we found that fed on post-sterilization bamboo leaves caused significant weight reduction of 3rd instar larva (*P* < 0.05). Surprisingly, 3rd instar larvae that were fed on-year leaves were significantly heavier than those fed off-year leaves (42.5% heavier; *P* < 0.01), but no difference was observed between the weights of 3rd instar larva fed sterilized on- and off-year leaves. These results suggest that microbes not only influence the pest resistance of moso bamboo to *P. phyllostachysae*, but also important to the development of larva.

### Gut microbial community structure and function of *P. phyllostachysae* feed on different types of bamboo leaves

**Fig. 2:**
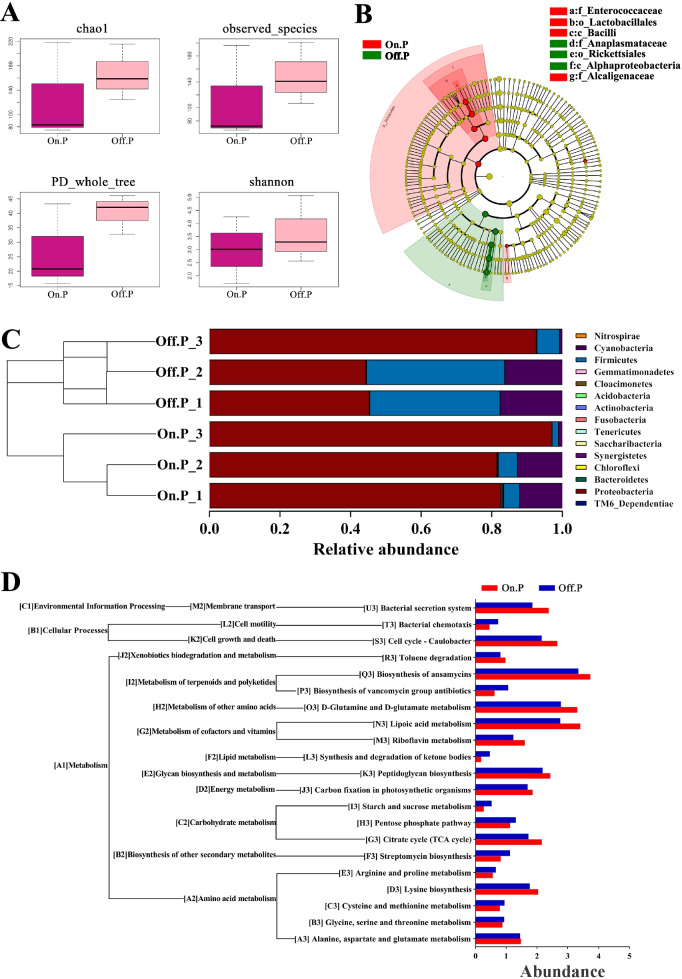
Gut microbiota of 3rd instar *Pantana phyllostachysae* larva fed on different types of moso bamboo leaves. **A** Microbial diversity of intestinal microbiota of *P. phyllostachysae* fed on on- (On.P) and off-year (Off.P) moso bamboo leaves were compared, and shown as the following indexes: Chao1, observed species, PD_whole_tree, and Shannon. The chao1 index represents the species richness index. Observed species index represent the number of OTUs observed as the sequencing depth increases. PD_whole_tree index is the pedigree diversity, which is a diversity index calculated based on the phylogenetic tree. The larger the value, the higher the community diversity. Shannon index is used to estimate the microbial diversity index in the sample. **B** The evolutionary branch diagram of LEfSe analysis based on Taxonomical information compared among different samples. The circle radiating represents the taxonomic level (from phylum to species) from inside to outside. Each small circle represents one taxonomic branch in the represented level and the diameter of the small circle is correlated with the relative abundance. The names of species are listed in the top-right. **C** Cluster histogram representing the relative richness of samples at the level of phylum. **D** Predicted functions of intestinal microbiota in *P. phyllostachysae*

The gut microbes of 3rd instar *P. phyllostachysae* larva including 15 phyla, 29 classes, 54 orders, 90 families, and 138 genera, and 457 OTUs were generated. The variations of gut microbiota between and within each species were measured by alpha diversities (Fig. 2A). The gut microbiota of *P. phyllostachysae* that were fed off-year moso bamboo leaves was higher in chao1 and observed species richness values, as well as PD whole tree and Shannon diversity indices. This result shows that the gut microbial diversities of the 3rd instar *P. phyllostachysae* larva fed on off-year bamboo leaves (Off.P) were higher than those fed on on-year bamboo leaves (On.P). The relative abundances of 10 major phyla (Proteobacteria, Cyanobacteria, Firmicutes, Bacteroidetes, Actinobacteria, Fusobacteria, Tenericutes, Chloroflexi, Cloacimonetes and Saccharibacteria) were commonly observed across all the samples. Linear discriminant analysis Effect Size (LEfSe) analysis was conducted to further explore the microorganisms that function in different types of gut microbiota [33] (Fig. 2B). We found that species of the families *Enterococcaceae* and *Alcaligenaceae*, order Lactobacillales, and class Bacilli have higher richness in On.P, while family *Anaplasmataceae*, order Rickettsiales, and class Alphaproteobacteria have higher richness in Off.P. At the phylum level (Fig. 2C), samples mainly contained members from Proteobacteria (73.87 ± 23.32% of total species), Firmicutes (15.69 ± 17.41% of total species) and Cyanobacteria (10.11 ± 7.41% of total species). Proteobacteria is the dominant phylum in the gut of *P. phyllostachysae* especially in those fed on-year bamboo leaves. We further analyzed the function of the gut microbial community and found that differential microbes were assigned to different functional groups (Fig. 2D). The different functional groups between On.P and Off.P include differences in A1 (metabolism), B1 (cellular processes), and C1 (environmental information processes). Differences in the main metabolism functional features include differences in: Q3 (Biosynthesis of ansamycins), O3 (D-Glutamine and D-glutamate metabolism), and N3 (Lipoic acid metabolism). These results show that the functions of microbial communities are closely related to the metabolism pathway of *P. phyllostachysae*.

### Functional microbe community of *P. phyllostachysae* larva in response to different diet

**Fig. 3:**
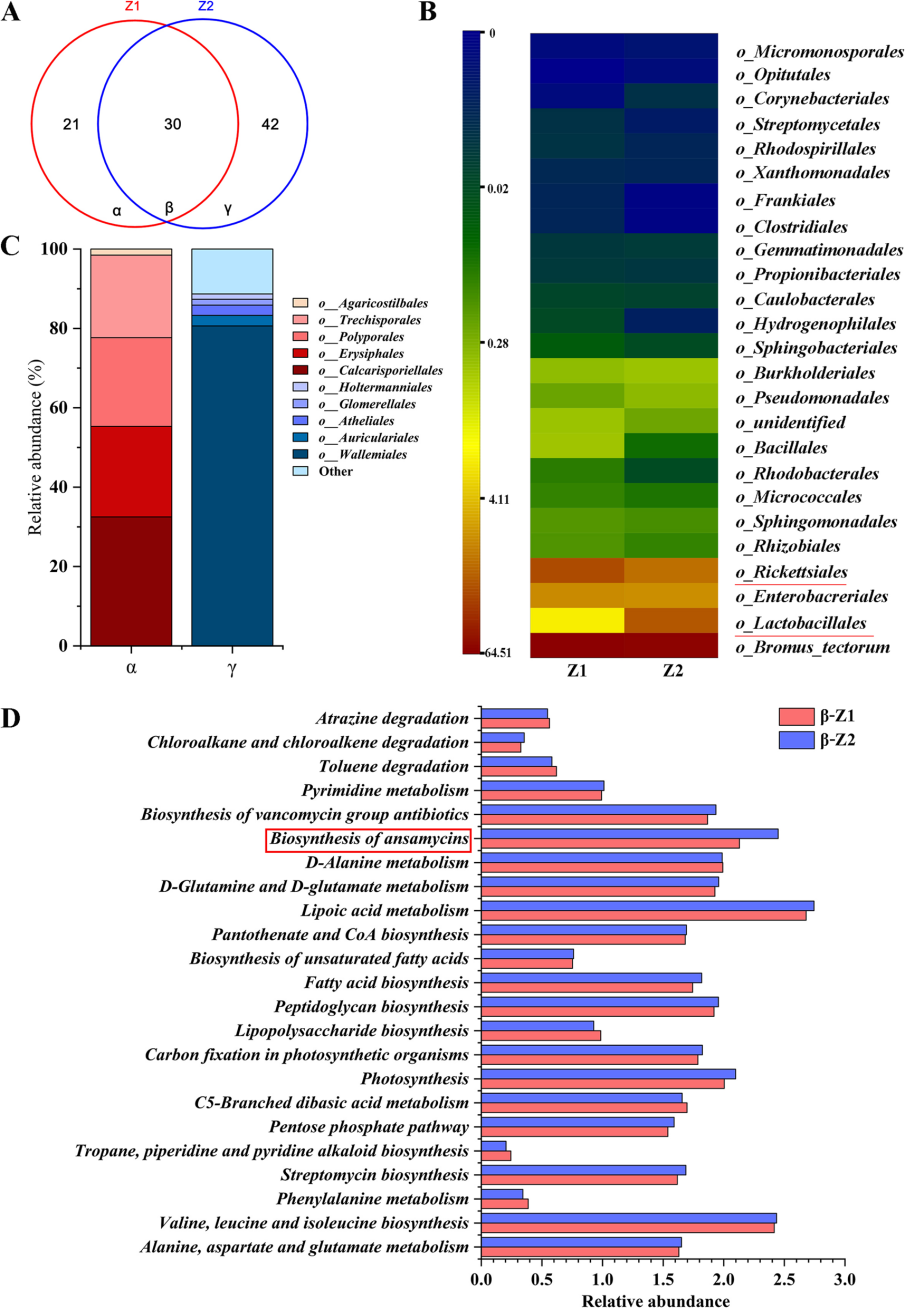
Functional microbiota of *Pantana phyllostachysae* larva in response to different diet. **A** Venn diagram of community structure of Z1 (mutual gut microbial community of on-year bamboo leaves and *P. phyllostachysae* fed on on-year bamboo leaves, excluding microbes present in *P. phyllostachysae* fed on post-sterilization leaves) and Z2 (mutual gut microbial community of off-year bamboo leaves and *P. phyllostachysae* fed on off-year bamboo leaves, excluding microbes present in *P. phyllostachysae* fed on post-sterilization leaves). β represents the common OTUs between Z1 and Z2, α represents the OTUs unique to Z1 compared to Z2, and γ represents the OTUs unique to Z2 compared to Z1. The microbial structures of β (**B**) on the level of order, and α and γ, on the level of genus (**C**) were compared. The function of β was plotted (**D**)

To further explore the functional microbiota in the gut of *P. phyllostachysae* larva related to moso bamboo resistance, metagenomic analysis of the gut of 3rd instar larva fed on pre- and post-sterilization leaves was conducted. The gut microbes of 3rd instar larva fed on post-sterilization leaves were excluded, then, the mutual species of on- and off-year bamboo leaves and the gut microbes of 3rd instar larva fed on them were assigned to Z1 (mutual gut microbial community of on-year bamboo leaves and *P. phyllostachysae* fed on on-year bamboo leaves, excluding microbes present in *P. phyllostachysae* fed on post-sterilization leaves) and Z2 (mutual gut microbial community of off-year bamboo leaves and *P. phyllostachysae* fed on off-year bamboo leaves, excluding microbes present in *P. phyllostachysae* fed on post-sterilization leaves), respectively (Fig. S3). Z1 was composed of 51 OTUs belonging to 9 phyla, 21 classes, 40 orders, 54 families, and 40 genera, while Z2 includes 72 OTUs belonging to 10 phyla, 21 classes, 40 orders, 49 families, and 42 genera. 30 OTUs (β) were shared by Z1 and Z2 (Fig. 3A) but only two out of these 35 orders, Lactobacillales and Rickettsiales, have significant differences between Z1 and Z2 (Fig. 3B). There are 21 and 42 OTUs in the specific microbial communities of Z1 (α) and Z2 (γ), respectively. Thermoanaerobacterales, Anaerolineales and Picea_glauca_white_spruce are the dominant orders in α, accounting for 40, 33 and 18% of the total specific species, respectively. While Nitrospirales has the highest abundance in γ, accounting for 43% of the total specific species (Fig. 3C). Furthermore, the functions of the microbial communities Z1 and Z2 were predicted. For microbes shared by Z1 and Z2, only the functional group of biosynthesis of ansamycins had significant difference between samples (Fig. 3D). For specific microbes in Z1 compared to Z2 (α), the dominant functional group was naphthalene degradation, with an abundance of 27%, while other functional groups had abundances less than 2%. As for γ, the dominant functional groups were polyketide sugar unit biosynthesis, metabolism of xenobiotics by cytochrome P450, and tetracycline biosynthesis, with abundances of 29, 28, and 26%, respectively (Fig. S4).

## Discussion

The biennial cycle of moso bamboo forms the different arthropod communities of on- and off-year bamboo leaves through bottom-up effect, which limits pest outbreaks in natural bamboo forests [2, 10, 11, 34]. However, IFM created ideal conditions for *P. phyllostachysae*, a destructive Lepidopteran pest that damages the largest area of bamboo forests annually. The present study investigated the effects of different host diets (on- and off-year bamboo leaves) on the taxonomic diversity, structure, and function of *P. phyllostachysae* gut microbiota. This provided an important insight of microbial efforts on the bottom-up effect of the “bamboo- *P. phyllostachysae*” nutrient system, however knowledge on the effects of biennial cycle on insets by microbes is still limited [35].

Developing an effective strategy to narrow down the functional microbes from microbiota is crucial for microbiome research [20]. In this study, we developed an efficient leaf sterilization assay to randomly eliminate the microbes on the bamboo leaves, which is capable of removing 88% of the total phyla from leaves without disturbing the leaf consumption of the larvae (Fig. S5). By utilizing the sterilization assay, the functional microbe community was narrowed down to less than 10% of the total differential gut microbiota of *P. phyllostachysae*. Thus, this study provided a new approach for characterizing host-microbial communities in leaf-boring moths and supplemented the information of insect’s microbiota in lepidopteran insects.

The resistance of host plant to its pests are considering as an integration of genetic responses, metabolites and microbes [13–15]. After removing the functional microbiota from moso bamboo leaves, the resistance to *P. phyllostachysae* was considered lost, with no difference observed between the weights of 3rd instar larva fed on different post-sterilized leaves (Fig. 1). This demonstrates that the microbes in different types of bamboo leaves are sufficient to impact *P. phyllostachysae* development. Meanwhile, obvious weight reduction of 3rd instar larva was observed by feeding on post-sterilized leaves, indicates that microbes are also essential to promote *P. phyllostachysae* development. Previously, with the help of RNA-seq, we can only partly explore the different response strategies of on- and off-year bamboo to the *P. phyllostachysae* attack, which is tolerance and offensive to the insect feeding respectively [13, 21]. Present study shows that microbes are extremely important to explain the different pest-resistance strategies of on- and off-year bamboo.

Although significant differences in microbial community diversity and structure were found between different types of bamboo leaves, no obvious differences of the leaf microbiotas were observed though the functional analysis of microbial communities. This irrelevant result may come from the limits of PICRUST2. Although PICRUST2 is an acceptable method for microbiota functional prediction, but do not correspond to gene detection or gene expression data, thus PICRUST2 predictions alone cannot be interpreted as evidence of presence or absence of metabolic functions [36], further experimentation and data on actual gene expression are required to verify the predictions. However, by comparing with the gut microbiota of *P. phyllostachysae*, we found that most dominant species in bamboo leaves cannot survive in the guts of the insects. This suggests that only a small portion of the leaf microbes function as pest-resistant agents [18]. The microbial communities of moso bamboo leaves impact metabolisms, which can explain the metabolomic differences between on- and off-year bamboo leaves [21].

Z1 and Z2 have structural differences at different taxonomic levels, especially at the order level. Orders Lactobacillales and Rickettsiales have the largest population within the gut microbial communities of different larva (Fig. 2B). The microbes within orders Lactobacillales and Rickettsiales are also the dominant species in the mutual group of the functional microbiota (β) with extremely significant differences (Fig. 3B). These findings indicate that, microbes of the orders Lactobacillales and Rickettsiales in the gut microbiota of *P. phyllostachysae* are crucial to the resistance of moso bamboo leaves to larva feeding. The abundance of order Lactobacillales is larger in the gut microbial communities of larva fed on on-year leaves. Lactobacillales is a bacillus-free bacterium which plays an important role in regulating the balance of intestinal microenvironments and inhibiting the growth of pathogenic bacteria by competing for nutrition, leading to the protection of the host insects [36]. Our findings indicate that one of the reasons *P. phyllostachysae* benefit from feeding on on-year bamboo leaves is that they contain microbes from the order Lactobacillales*.* On the contrary, microbes of order Rickettsiales had greater richness in the guts of larva fed on off-year leaves. Rickettsiales can kill the host insects by restricting the developmental speed, lowering the environmental fitness [37, 38], and limiting resistance to pathogenic bacteria and natural enemies [39, 40]. These findings agree with our previous work, which found that the microbes in off-year leaves limit the development of *P. phyllostachysae* compared to on-year. These results suggests that microbes of the orders Lactobacillales and Rickettsiales can be a powerful microbial pesticide for *P. phyllostachysae* larva.

For common functional gut microbiota, the functional group (biosynthesis of ansamycin) of β holds greater richness in Z2 than Z1 (Fig. 2D), leading to the functional microbiota more heavily disturbing the balance of the intestinal microenvironments of larva fed on off-year leaves compared to on-year leaves [41]. For specific functional gut microbiota (Fig. S4), larva fed on on-year leaves can benefit from the dominant microbial species within α which function for naphthalene degradation [42]. However, the dominant microbial species within γ have functions for polyketide.

sugar unit biosynthesis and metabolism of xenobiotics by cytochrome P450, which can severely damage the immune system of host insects [43, 44]. Some of the dominant microbial species have functions for tetracycline biosynthesis, which is capable of disturbing the balance of intestinal microenvironment [45].

## Conclusions

The present work is the first to demonstrate that microbes lead to differences in *P. phyllostachysae* larva that are fed different diets (on- and off-year moso bamboo leaves). We used a newly developed random artificial assemble approach (leaf sterilization assay). We also explored the microbial traits by metagenomic sequencing of guts of 3rd instar *P. phyllostachysae* larva fed different diets (pre- and post-sterilized leaves), and we found species from the orders Lactobacillales and Rickettsiales to be responsible for the greatest differences in larva development. By utilizing microbial function prediction, we hypothesized that most functional microbes in larva that feed on on-year bamboo leaves are beneficial to their host, whereas the functional microbes in larva that feed on off-year bamboo leaves are detrimental to the balance of their intestinal microenvironments and immune systems. This work provided critical insight into functional microbiota that affect *P. phyllostachysae* development with respect to its feeding behavior, furthermore, it will be more meaningful that investigate the dynamics of microbiota in the gut of *P. phyllostachysae* that feed on bamboo leaves grown during different periods of treatments and concentration of the antibiotic to further narrow down the identities of the functional microbes. In addition, due to the debatable accuracy of gene function prediction analysis after 16 s sequencing, further microbial function analysis and verification are required to identify the mechanism underlying pest outbreaks in commercial moso bamboo forests. We are currently isolating the candidates from the orders Lactobacillales and Rickettsiales in leaf and gut microbiota and further investigating the efficiency of these biocontrol agents in the field.

## Supplementary Information

**Additional file 1: Fig. S1.** Microbiota on different types of moso bamboo leaves. **Fig. S2.** Efficiency of the leaf sterilization assay. **Fig. S3.** Illumination of Z1 and Z2. **Fig. S4.** Functional prediction of specific microbial communities within Z1 and Z2.

## Data Availability

The datasets generated and/or analysed during the current study are available in the NCBI database (National Center for Biotechnology Information; BioProject ID: PRJNA778056), also included within the article (and its additional files).

## Abbreviations

IFM: Intensive forest management
VOCs: Volatile organic compounds
PGPF: Plant growth-promoting fungi
PGPR: Plant growth-promoting rhizobacteria
OTUs: Operational taxonomic units
ASV: Amplicon sequence variant
COG: Cluster of Orthologous Groups
On.P: The gut microbial diversities of the 3rd instar *P. phyllostachysae* larva fed on on-year bamboo leaves
Off.P: The gut microbial diversities of the 3rd instar *P. phyllostachysae* larva fed on off-year bamboo leaves
LEfSe: Linear discriminant analysis Effect Size
Z1: Mutual gut microbial community of on-year bamboo leaves and *P. phyllostachysae* fed on on-year bamboo leaves, excluding microbes present in *P. phyllostachysae* fed on post-sterilization leaves
Z2: Mutual gut microbial community of off-year bamboo leaves and *P. phyllostachysae* fed on off-year bamboo leaves, excluding microbes present in *P. phyllostachysae* fed on post-sterilization leaves
On.B: On-year bamboo leaves
Off.B: Off-year bamboo leaves
