# Supplementary material for: The functional microbiota of on- and off-year moso bamboo (Phyllostachys edulis) influences the development of the bamboo pest Pantana phyllostachysae
Source: BMC Plant Biol. 2022 Jun 24;22:307. doi: 10.1186/s12870-022-03680-z (PMC9229751; doi:10.1186/s12870-022-03680-z)
Supplement: Supplementary file 1 — Additional file 1: Fig. S1. Microbiota on different types of moso bamboo leaves. Fig. S2. Efficiency of the leaf sterilization assay. Fig. S3. Illumination of Z1 and Z2. Fig. S4. Functional prediction of specific microbial communities within Z1 and Z2. [file 12870_2022_3680_MOESM1_ESM.pdf]

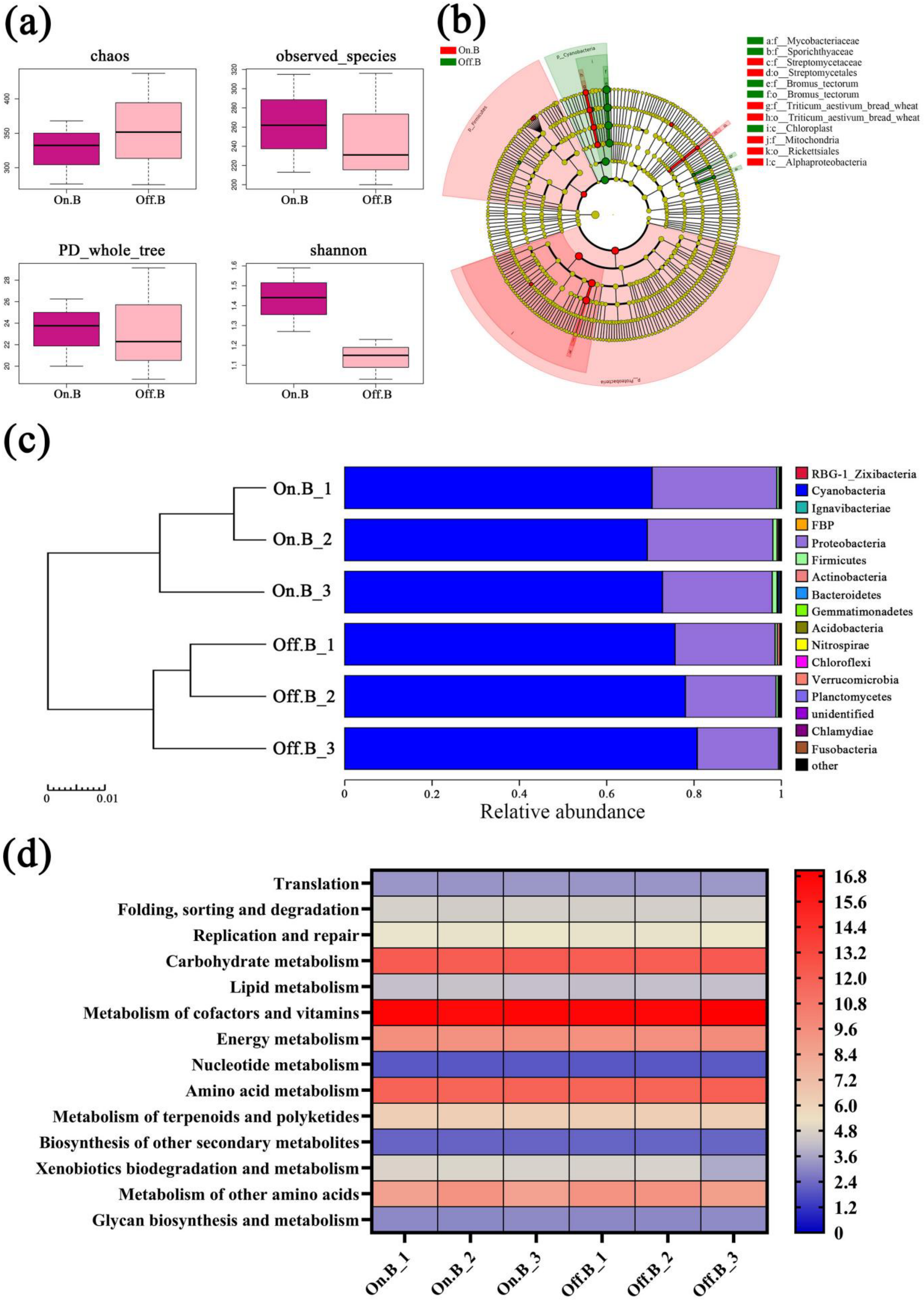

(a)
chaos
On.B
Off.B
observed_species
On.B
Off.B
PD_whole_tree
On.B
Off.B
shannon
On.B
Off.B
(b)
On.B
Off.B
a:f__Mycobacteriaceae
b:f__Sporichthyaceae
c:f__Streptomycetaceae
d:o__Streptomycetales
e:f__Bromus_tectorum
f:o__Bromus_tectorum
g:f__Triticum_aestivum_bread_wheat
h:o__Triticum_aestivum_bread_wheat
i:c__Chloroplast
j:f__Mitochondria
k:o__Rickettsiales
l:c__Alphaproteobacteria
(c)
On.B_1
On.B_2
On.B_3
Off.B_1
Off.B_2
Off.B_3
0 0.01
0 0.2 0.4 0.6 0.8 1
Relative abundance
RBG-1_Zixibacteria
Cyanobacteria
Ignavibacteriae
FBP
Proteobacteria
Firmicutes
Actinobacteria
Bacteroidetes
Gemmatimonadetes
Acidobacteria
Nitrospirae
Chloroflexi
Verrucomicrobia
Planctomycetes
unidentified
Chlamydiae
Fusobacteria
other
(d)
Translation
Folding, sorting and degradation
Replication and repair
Carbohydrate metabolism
Lipid metabolism
Metabolism of cofactors and vitamins
Energy metabolism
Nucleotide metabolism
Amino acid metabolism
Metabolism of terpenoids and polyketides
Biosynthesis of other secondary metabolites
Xenobiotics biodegradation and metabolism
Metabolism of other amino acids
Glycan biosynthesis and metabolism
On.B_1
On.B_2
On.B_3
Off.B_1
Off.B_2
Off.B_3
16.8
15.6
14.4
13.2
12.0
10.8
9.6
8.4
7.2
6.0
4.8
3.6
2.4
1.2
0

Fig. S1 Microbiota on different types of moso bamboo leaves. (a) Microbial diversities of on- (On.B) and off-year (Off.B) moso bamboo leaves were compared, and shown as chao1, observed_species, PD_whole_tree, and Shannon. (b) The evolutionary branch diagrams of LEfSe analysis based on taxonomical information were compared among different samples. The circle radiating represents the taxonomic level (from phylum to species) from inside to outside. Each small circle represents one taxonomic branch in the represented level, and the diameter of the small circle is correlated with the relative abundance. The name of the species are listed in the top-right. (c) Cluster histograms representing the relative richness of samples at the level of phylum. (d) Predicted functions of intestinal microbiota of on- (On.B) and off-year (Off.B) moso bamboo leaves. The boxes indicate the number of moso bamboo leaves microbial species enriched in each functional group.

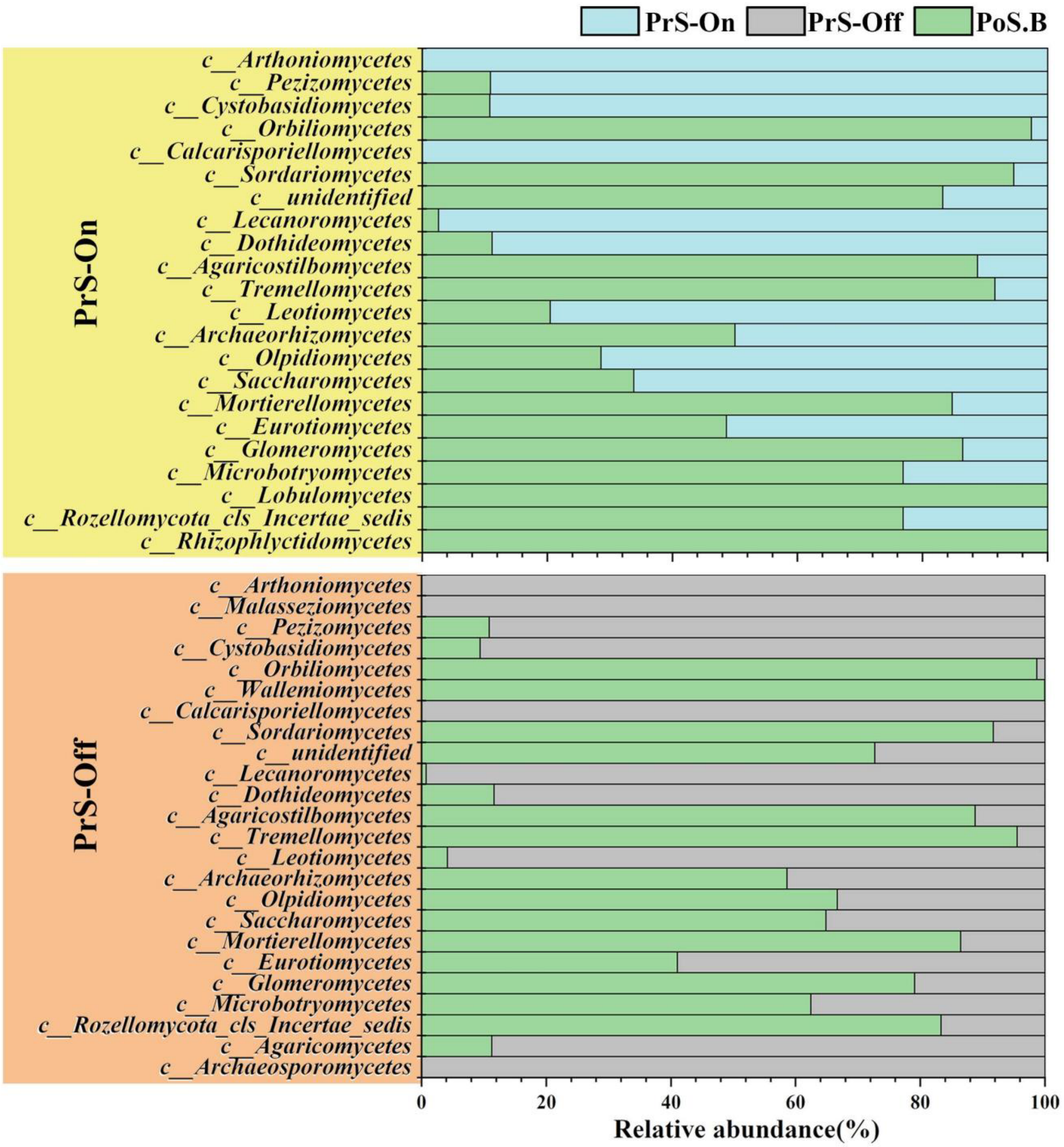

Fig. S2 Efficiency of the leaf sterilization assay. The sterilization efficiencies of on- and off-year bamboo leaves were compared by OTUs in different classes of the pre-sterilized on- (PrS-On) and off-year (PrS-Off) bamboo leaves, as well as post-sterilized bamboo leaves (PoS.B).

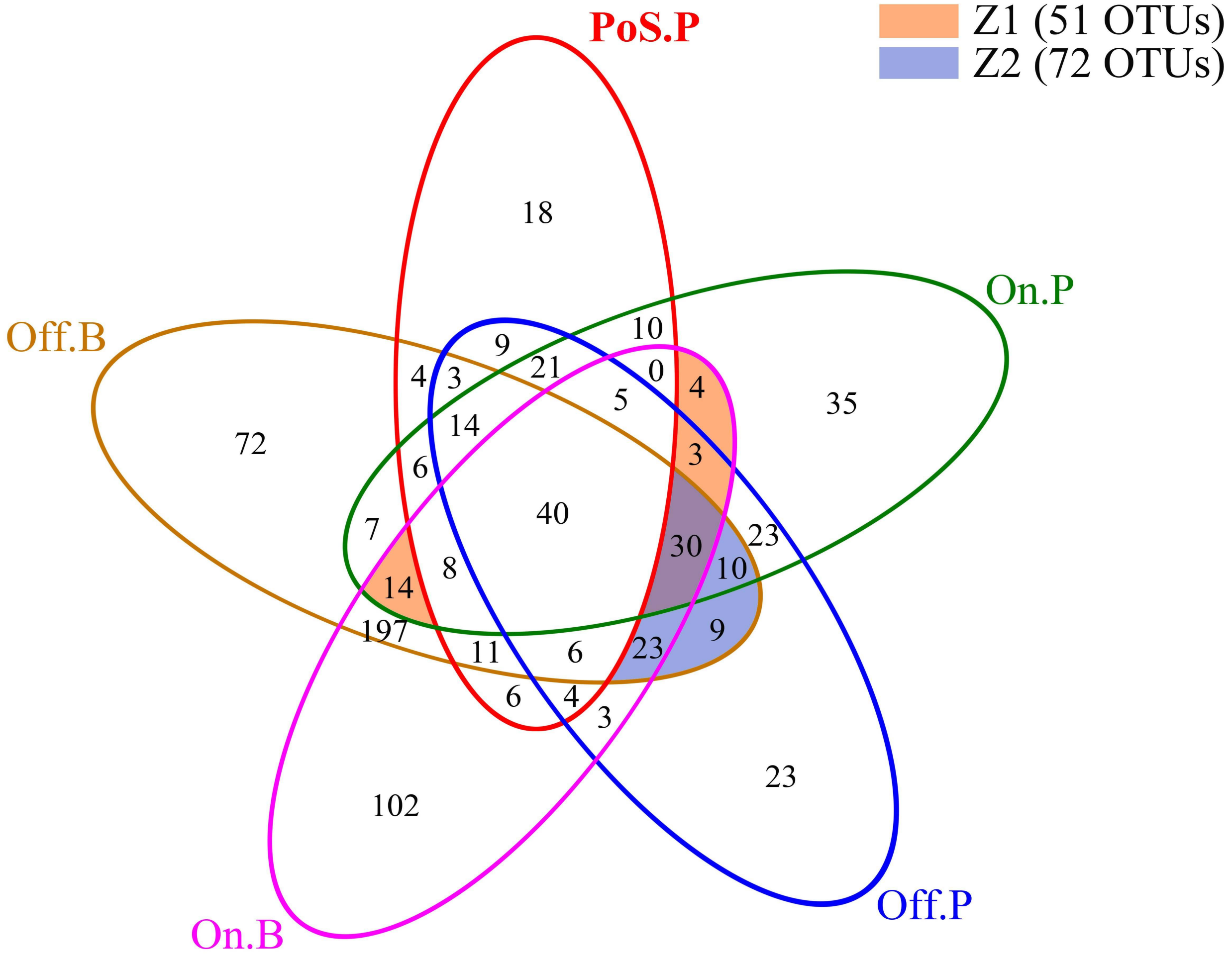

Fig. S3 Illumination of Z1 and Z2. Venn diagram of OTU numbers of microbial communities of different samples. On.B stands for on-year bamboo leaves, Off.B stands for off-year bamboo, On.P stands for *P. phyllostachysae* fed on on-year bamboo leaves, Off.P stands for *P. phyllostachysae* fed on off-year bamboo leaves, and PoS.P represent *P. phyllostachysae* fed on autoclaved bamboo leaves.

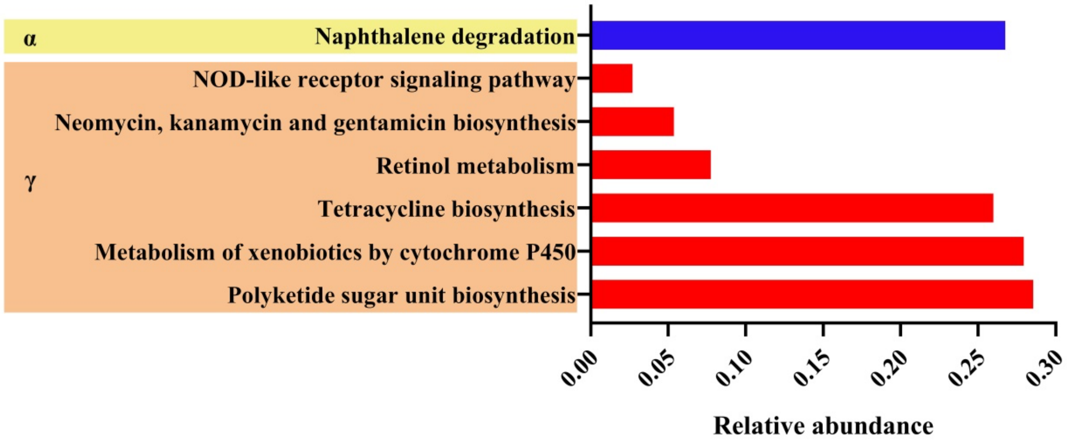

Fig. S4 Functional prediction of specific microbial communities within Z1 and Z2. α represents the specific OTUs in Z1 compared to Z2, and γ represents the specific OTUs in Z2 compared to Z1.
